# When to use a prophylactic mesh after stoma closure: a case–control study

**DOI:** 10.1007/s10029-021-02508-3

**Published:** 2021-11-12

**Authors:** C. Ramírez-Giraldo, A. Torres-Cuellar, C. Cala-Noriega, C. E. Figueroa-Avendaño, J. Navarro-Alean

**Affiliations:** 1 Universidad del Rosario, Carrera 24 #63C - 69, Bogotá, Colombia; 2 Hospital universitario Mayor – Méderi, Bogotá, Colombia

**Keywords:** Incisional hernia, Stoma, Parastomal hernia, Mesh

## Abstract

**Purpose:**

The closure of a stoma is frequently associated with an acceptable morbidity and mortality. One of the most frequent complications is incisional hernia at the stoma site, which occurs in 20%–40% of cases, higher than incisions in other parts of the abdomen. The objective of this study was to identify the risk factors associated with the presentation of incisional hernia after stoma closure, this in order to select patients who are candidates for prophylactic mesh placement during closure.

**Methods:**

An unpaired case–control study was conducted. This study involved 164 patients who underwent a stoma closure between January 2014 and December 2019. Associated factors for the development of incisional hernia at the site of the stoma after closure were identified, for which it was performed a logistic regression analysis.

**Results:**

41 cases and 123 controls were analyzed, with a mean follow-up of 35.21 ± 18.42 months, the mean age for performing the stoma closure was 65.28 ± 14.07 years, the most frequent cause for performing the stoma was malignant disease (65.85%). Risk factor for the development of incisional hernia at the stoma site after its closure was identified as a history of parastomal hernia (OR 5.90, CI95% 1.97–17.68).

**Conclusions:**

The use of prophylactic mesh at stoma closure should be considered in patients with a history of parastomal hernia since these patients present a significantly higher risk of developing a hernia.

## Introduction

The closure of a stoma is a procedure that is executed frequently related to acceptable morbidity and mortality [1]. One of the most frequent complications, which is occasionally underestimated, is the incisional hernia in the location of the stoma, which occurs in 20%–40% of cases, being higher than other incisions in other parts of the abdomen [2, 3]. Its presence can cause abdominal pain, discomfort and decrease the quality of life [4, 5].

Multiple risk factors have been described for the development of a hernia after the closure of a stoma such as the female sex, high body mass index, a stoma prolapse, parastomal hernia, hypertension, closure of colostomy in malignant disease, mid-line hernia, and postoperative complications [4–8].

The correction of an incisional hernia after the closure of the stoma may be a challenge due to the adhesions of the previous procedure and the comorbidities of the patients; additionally, it may arise in an emergency instance such as in incarcerated or strangulated hernia [2]. One of the options to decrease the incidence and avoid a second procedure for the correction of the hernia is the placement of a prophylactic mesh [2, 8]. However, employing a mesh may arise other complications such as an infection in the surgical site, increasing surgical time, and increasing costs [2].

The prophylactic meshes have been proven in diverse studies to decrease the rate of appearance of incisional hernias after the closure of the stoma; the issue is to state which patient benefits from a prophylactic mesh according to their risk factors if we appropriately define the patient that would benefit from a prophylactic mesh and those who would not, we would be adequately managing resources and reduce morbidity and mortality of an additional procedure if it is not necessary [1, 9–11].

This study aims to identify the factors associated with the appearance of an incisional hernia, to select patients that would benefit from the placement of a prophylactic mesh after the closure of a stoma.

## Patients and methods

### Study design

Between January 2014 and December 2019, all the patients who underwent a stoma were identified through a database gathered prospectively. The database had a total of 433 patients. We reviewed the database until we reached the calculated sample. A study of unmatched cases-controls was performed, this study included 164 patients. The factors associated with the development of an incisional hernia in the site of the stoma after its closure were identified. This study was revised and approved by the Ethics Committee of Universidad del Rosario. We followed the STROBE guidelines to report this study [13].

### Patients

We define a case as the patient who, during follow-up, presented with a hernia on the stoma closure site. We define control as a patient who showed no evidence of a hernia on the location of stoma closure site during follow-up.

We included patients over 18 years old who underwent stoma closure (loop or end ileostomy or colostomy), due to benign or malignant disease. We excluded patients who required a new stoma after the closure of the first stoma in the same site of the abdominal wall; we also excluded the patients who were dropped during the monitoring.

We analyzed the following data: demographic characteristics of the patients, body mass index, ASA Physical Status Classification, presence of diabetes mellitus, chronic obstructive pulmonary disease (COPD), coronary disease, history of smoking, presence of malignant disease, neoadjuvant or adjuvant treatment (including radiotherapy and/or chemotherapy), the indication of surgical procedure, type of surgical procedure (open or laparoscopic surgical procedure), surgical variables (material and technique of suture for the closure of the aponeurosis), primary resection type, stoma-related complication, characteristics of the stoma (colostomy or ileostomy, loop or end), complications related to the surgical procedure, and duration of follow-up.

The presence of an incisional hernia in the site of closure of the stoma was assessed through a physical exam with the patient in a supine position and standing position.

### Surgical procedure

There were multiple reasons to indicate performing the stoma, such as anastomotic dehiscence, intestinal obstruction, protection stomas or intestinal perforation where, due to the cavity's contamination or hemodynamic instability, it was decided that a stoma should be performed.

The institutional protocol for stoma closure depends on the situation due to which it was conducted. In the case of patients with protective stomas, their stoma was closed once there was evidence that the anastomosis had healed completely, that the adjuvant treatment had been completed if it had been ordered and that they were in a disease-free period in the cases of malignant disease. In the case of a patient with an end stoma, we waited for a minimum period of 4 months from the performance of the procedure; we also waited for the adjuvant treatment to finish in the cases where it had been ordered. In all cases where the closure of the soma was selected, the patients had adequate functioning and an adequate nutritional status. Before closing the stoma, all patients always underwent an imaging study (barium enema or abdominal CT scan) and an endoscopic study.

All stoma closures were performed using mechanical suture devices. For loop stomas, a localized closure was performed; for end stomas, they were done with laparoscopy when possible, and when not, with laparotomy. The aponeurotic defect where the stoma was closed with polydioxanone and continuous or interrupted suture according to the surgeon’s preference, and the skin closure was a delayed primary closure in all cases to avoid surgical site infections.

### Statistical analysis

A sample size of 41 cases and 123 controls was calculated, resulting in a total of 164 patients. A 95% confidence interval and 80% power, a 20% of cases exposure, an OR of three to detect the difference in any risk factor and a relation 1:3 per case–control were considered.

The categorical variables are provided as percentages and were contracted through the Fisher’s exact test or Chi squared test and the continuous variables were contrasted through the Mann–Whitney *U* test or Test *T*, as applicable. A multivariate analysis was performed to identify the independent factors for the development of an incisional hernia. The variables were included with *p* < 0.1 in the univariate analysis and those considered as clinically relevant.

The entire analysis was performed in Epidat 4.2, considering a statistically significant *p* < 0.05.

## Results

Our institution performs on average 73 stoma closures per year, and we have a documented incidence of hernia after stoma closure of 22.7% of cases.

41 cases and 123 controls were analyzed, with average monitoring of 35.21 ± 18.42 months, the age average for the performance of a stoma was 65.28 ± 14.07 years, and the most frequent cause to perform it was a malignant disease [65.85%]. Other demographic, clinical, and surgical characteristics are detailed in Table 1.

**Table 1:** Demographic characteristics, clinical characteristics, and surgical factors of the patients who underwent a stoma

	Total *n* = 164	No hernia (%) *n* = 123 (75)	Hernia (%) *n* = 41 (25)	Value *p*
Age (mean ± SD) (years)	65.28 (14.07)	63.99 (14.23)	69.14 (12.98)	**0.042**
Sex				**0.787**
Female	85 (51.83)	63 (38.41)	22 (13.41)	
Male	79 (48.17)	60 (36.58)	19 (11.58)	
ASA classification				**0.088**
1	46 (28.05)	39 (23.7)	7 (4.27)	
2	117 (71.34)	83 (50.60)	34 (20.73)	
3	1 (0.61	1 (0.61)	0 (0)	
04-May	0	0 (0)	0 (0)	
Body mass index (mean ± SD) (kg/m^2^)	25.35 (3,90)	25.29 (3.94)	25.52 (3.82)	**0.745**
Indication for stoma construction				
Protection of anastomosis	55 (33.54)	43 (26.21)	12 (7.31)	**0.504**
Anastomotic leak	2 (1.22)	1 (0.61)	1 (0.61)	**0.411**
Acute colonic obstruction	69 (42.07)	51 (31.10)	18 (10.98)	**0.784**
Diverticulitis	12 (7.32)	8 (4.88)	4 (2.44)	**0.489**
Other	26 (15.85)	20 (12.2)	6 (3.66)	**0.805**
Current smoker	29 (17.68)	21 (12.8)	8 (4.88)	**0.723**
Co-morbidity				
Hypertension	50 (30.49)	37 (22.56)	13 (7.93)	**0.845**
Diabetes mellitus	27 (16.46)	20 (12.2)	7 (4.27)	**0.903**
COPD	6 (3.66)	5 (3.05)	1 (0.61)	**0.631**
Cardiovascular disease	11 (6.71)	7 (4.27)	4 (2.44)	**0.368**
Underlying malignant disease	108 (65.85)	82 (50)	26 (15.85)	**0.704**
Primary abdominal surgery				**0.467**
Laparoscopic	92 (56.1)	67 (40.85)	25 (15.24)	
Open	72 (43.9)	56 (34.1)	16 (9.76)	
Primary resection type				
Left hemicolectomy	18 (10.98)	13 (7.93)	5 (3.05)	**0.773**
Right hemicolectomy	39 (23.78)	27 (16.46)	12 (7.32)	**0.341**
Subtotal colectomy	14 (8.54)	13 (7.93)	1 (0.61)	**0.107**
Sigmoidectomy	44 (26.83)	32 (19.5)	12 (7.32)	**0.684**
Low anterior resection	46 (28.05)	37 (22.56)	9 (5.49)	**0.316**
Adjuvant therapy	84 (51.22)	61 (37.2)	23 (14.02)	**0.471**
Neoadjuvant therapy	31 (18.9)	29 (17.68)	4 (2.44)	**0.056**
Type of stoma				**0.237**
Ileostomy	93 (56.71)	73 (44.51)	20 (12.2)	
Colostomy	71 (43.29)	50 (30.49)	21 (12.8)	
Type of stoma				**0.63**
End	111 (67.68)	82 (50)	29 (17.68)	
Loop	53 (32.3)	41 (25)	12 (7.32)	
Stoma-related complication				
Parastomal hernia	18 (10.98)	7 (4.27)	11 (6.71)	** < 0.001**
Prolapse	17 (10.37)	14 (8.54)	3 (1.83)	**0.46**
High output	6 (3.66)	5 (3.05)	1 (0.61)	**0.631**
Obstruction	0	0	0	
Necrosis	0	0	0	
Retraction	0	0	0	
Suture technique				**0.121**
Continuous	112 (68.29)	80 (48.78)	32 (19.51)	
Intermittent	52 (31.71)	43 (26.22)	9 (5.49)	
Stoma closure technique				
Local reversal	81 (49.39)	58 (35.37)	23 (14.02)	**0.321**
Including laparotomy	60 (36.59)	49 (29.88)	11 (6.71)	**0.134**
Including laparoscopy	23 (14.02)	16 (9.76)	7 (4.27)	**0.516**
Complications after reversal				
Surgical site infection	5 (3.05)	4 (2.44)	1 (0.61)	**0.793**
Anastomotic leak	5 (3.05)	4 (2.44)	1 (0.61)	**0.793**
Reintervention	13 (7.93)	12 (7.32)	1 (0.61)	**0.133**
Death	0	0	0	
Mid-line hernia	45 (27.44)	34 (20.73)	11 (6.71)	**0.92**
Duration of follow-up (mean ± SD) (months)	35.21 (18.42)	36.46 (18.79)	31.47 (16.93)	**0.116**
Stoma closure interval (mean ± SD) (months)	14,10 (10.03)	13.42 (10.45)	16.12 (8.45)	**0.136**

Values <0.1 were included in multivariate analysis.

The age, ASA Physical Status Classification, parastomal hernia, and neoadjuvant were identified as potential risk factors for the development of incisional hernias (p < 0.1 in the univariate analysis) therefore, were included in the multivariate analysis. The presence of a parastomal hernia was identified as an independent risk factor for the development of an incisional hernia (Table 2).

**Table 2:** Multivariate analysis of logistic regression of risk factors for the development of an incisional hernia after the closure of a stoma

	OR	IC (95%)
Age	1.19	0.98–1.05
ASA classification	1.69	0.63–4.48
Neoadjuvant therapy	0.31	0.09–1.02
Parastomal hernia	5.90	1.97–17.68

## Discussion

An incisional hernia after the closure of a stoma is a complication evidenced frequently [12]. After displaying this complication, the patient must undergo corrective surgery; however, this is not exempt or complications and of a relevant recurrence rate. Whereupon, it is necessary to identify patients with a higher propensity of developing a hernia to take preventive measures.

Within our study, the presence of a parastomal hernia was identified as the main risk factor for the development of incisional hernias after the closure of a stoma. This finding has been already stated in previous studies, where besides this finding, risk factors of obesity, hypertension, the presence of malignant disease, diabetes mellitus, and stoma prolapse were discovered [4, 6, 13]. Nevertheless, we were unable to identify any of the other described factors.

The parastomal hernia, as an identified risk factor, is presumably related to the presence of a higher aponeurotic defect with which the wall might have higher weakness and predispose the development of an incisional hernia.

One of the factors described in the literature is the presence of an end colostomy and end stoma due to the larger diameter of aponeurotic defect to perform the stoma [5]. Our study did not find statistically significant differences with respect to the type of stoma.

The skin closure technique on the stoma site has been described in different ways, such as secondary intention skin healing, several primary closure methods (“air-tight” primary closure, “loose” primary closure or “delayed” primary closure), or a hybrid method utilizing a purse-string suture. The reason for such a wide range of described techniques is that physicians wish to avoid surgical site infections due to the wound being contaminated with intestinal flora when closing the stoma. Surgical site infection is accepted as a risk factor for the development of incisional hernias [5]. We did not take this variable into account for our analysis because all patients underwent a delayed primary closure, which is part of our protocol.

The European Hernia Society guidelines on the closure of abdominal wall incisions do not include a recommendation regarding the surgical technique to employ when closing lateral aponeurotic defects due to the lack of evidence. Therefore, there is no recommendation for closing the aponeurosis on the site where the stoma was. However, we have extrapolated the recommendations for midline closure. We performed a suture to wound length ratio (SL/WL) of at least 4/1 for continuous closure, the “small bites technique” for continuous closure, used slowly absorbable suture material and monofilament suture material; the only recommendation we did not follow was using a continuous closure; a continuous or interrupted closure was used according to the surgeon's preference [16]. However, the analysis did not show statistically significant differences between the continuous and the interrupted closure of the aponeurosis.

Interest in the use of prophylactic mesh has been increasing. In spite of that, most surgeons do not use this option to avoid incisional hernias, due to multiple reasons such as weak supporting evidence (a weak recommendation under European Hernia Society guidelines), concern about its potential deleterious effects, and the fact that they are not convinced of its benefits [17]. Nonetheless, evidence of the use of prophylactic mesh has been increasing with the goal of breaking the vicious cycle created by the presence of an incisional hernia, complications and morbidity of its repair, the recurrence of that repair and further complications, and the ever-increasing difficulty of having a healthy abdominal wall [9]. In addition to the benefit for the patient, the costs of an incisional hernia are considerably high; therefore, preventing it would provide significant economic savings [5, 9, 18].

There are surgical preventive strategies in multiple pathologies, such as mastectomies and colectomies in patients at a high risk of cancer, or the insertion of urethral stents in colorectal surgery to identify urethral injuries. The prevention of incisional hernias should not be an exception considering the benefits, both economic and for the patient [9].

Currently, the trend is the employment of prophylactic meshes in the site of the closure of the stomas in all patients, which has demonstrated effectiveness with different types of meshes employed [2, 14]. In our setting and low-income countries, the cost of an additional procedure such as a prophylactic mesh may not be possible; hence it is necessary to select the patients more prone to present a hernia and offer them this additional procedure, which may be more cost-effective than the employment of a prophylactic mesh in all patients [5, 15].

The most relevant limitations of this study are their retrospective design, which makes it prone to selection biases. Certain controls could present small asymptomatic incisional hernias that went unnoticed, which incorrectly classified them as controls.

It is essential to perform a systematic review of the risk factors stated in new studies to determine which have more association to incisional hernias after the closure of the stoma and define in these patients, the employment of prophylactic meshes since they have been demonstrated as safe and effective in multiple studies [13, 16].

## Conclusion

The employment of a prophylactic mesh after the closure of a stoma must be considered for patients with a history of parastomal hernia since these patients have a higher risk to develop a hernia at the site of the stoma.

## Data Availability

Upon request.
